# Biomarkers for Assessing Non-Alcoholic Fatty Liver Disease in Patients with Type 2 Diabetes Mellitus on Sodium–Glucose Cotransporter 2 Inhibitor Therapy

**DOI:** 10.3390/jcm12206561

**Published:** 2023-10-16

**Authors:** Farah Khaznadar, Ana Petrovic, Omar Khaznadar, Hrvoje Roguljic, Kristina Bojanic, Lucija Kuna Roguljic, Stjepan Siber, Robert Smolic, Ines Bilic-Curcic, George Y. Wu, Martina Smolic

**Affiliations:** 1 Faculty of Dental Medicine and Health Osijek, Josip Juraj Strossmayer University of Osijek, 31000 Osijek, Croatia; farah.khaznadar.8@gmail.com (F.K.); anapetrovic@fdmz.hr (A.P.); hroguljic@mefos.hr (H.R.); kbojanic@fdmz.hr (K.B.); lkuna@fdmz.hr (L.K.R.); ssiber@fdmz.hr (S.S.); rsmolic@fdmz.hr (R.S.); 2 Faculty of Medicine Osijek, Josip Juraj Strossmayer University of Osijek, 31000 Osijek, Croatia; ibcurcic@mefos.hr; 3 Department of Radiology, “Dr. Juraj Njavro” National Memorial Hospital Vukovar, 32000 Vukovar, Croatia; khaznadar.omar@gmail.com; 4 Clinical Hospital Center, 31000 Osijek, Croatia; 5 Health Center Osijek-Baranja County, 31000 Osijek, Croatia; 6 Department of Medicine, Division of Gastrenterology/Hepatology, University of Connecticut Health Center, Farmington, CT 06030, USA; wu@uchc.edu

**Keywords:** SGLT2 inhibitors, NAFLD, biomarkers, diabetes mellitus type 2

## Abstract

In the current modern era of unhealthy lifestyles, non-alcoholic fatty liver disease (NAFLD) is the most prevalent liver disease and has become a serious global health problem. To date, there is no approved pharmacotherapy for the treatment of NAFLD, and necessary lifestyle changes such as weight loss, diet, and exercise are usually not sufficient to manage this disease. Patients with type 2 diabetes mellitus (T2DM) have a significantly higher risk of developing NAFLD and vice versa. Sodium–glucose cotransporter 2 (SGLT2) inhibitors are antidiabetic agents that have recently been approved for two other indications: chronic kidney disease and heart failure in diabetics and non-diabetics. They are also emerging as promising new agents for NAFLD treatment, as they have shown beneficial effects on hepatic inflammation, steatosis, and fibrosis. Studies in animals have reported favorable effects of SGLT2 inhibitors, and studies in patients also found positive effects on body mass index (BMI), insulin resistance, glucose levels, liver enzymes, apoptosis, and transcription factors. There are some theories regarding how SGLT2 inhibitors affect the liver, but the exact mechanism is not yet fully understood. Therefore, biomarkers to evaluate underlying mechanisms of action of SGLT2 inhibitors on the liver have now been scrutinized to assess their potential as a future in-label therapy for NAFLD. In addition, finding suitable non-invasive biomarkers could be helpful in clinical practice for the early detection of NAFLD in patients. This is crucial for a positive disease outcome. The aim of this review is to provide an overview of the most recent findings on the effects of SGLT2 inhibitors on NAFLD biomarkers and the potential of SGLT2 inhibitors to successfully treat NAFLD.

## 1. Introduction

The prevalence of non-alcoholic fatty liver disease (NAFLD), the most common liver disease, has been reported to be as high as 25%. It is characterized by the presence of fat in at least 5% of hepatocytes [1,2]. Lipid homeostasis in the liver is controlled by a very complex interplay between nuclear receptors, transcription factors, and hormones. Excessive fat storage occurs when the balance between lipogenesis/lipid uptake vs. lipid oxidation/excretion is disrupted [3,4]. NAFLD is usually associated with metabolic syndrome and is not, by definition, caused by excessive alcohol intake (defined as ≥20 g per day for women and ≥30 g per day for men) [1,2]. NAFLD represents a wide range of liver diseases, but histologically, it can be divided into two different types: non-alcoholic fatty liver (simple steatosis or NAFL) and non-alcoholic steatohepatitis (NASH), which is the more aggressive form [5,6,7]. Liver inflammation and injury are typical for NASH, and this form of NAFLD could lead to liver cirrhosis and hepatocellular cancer [2,8]. Due to the slow and asymptomatic progression of NAFLD, it is often diagnosed at a late stage. NAFLD has already become the second most common indication for liver transplantation. It takes around three to seven years for the development of NASH, which occurs in one-fifth of patients with NAFLD [9,10]. By the year 2027, it is expected that 18 million people in Japan, the US, and Europe (Germany, England, Italy, Spain, and France) will be affected by NASH [2]. Genetic predisposition also plays a role in NAFLD development, and the most common polymorphisms detected in NAFLD patients are genetic variations of transmembrane 6 superfamily member 2 (TM6SF2), patatin-like phospholipase domain-containing protein 3 (PNPLA3), and glucokinase regulatory protein (GCKR) [11].

A meta-analysis revealed that patients with type 2 diabetes mellitus (T2DM) have more than two-fold higher risk of developing serious hepatic disease [12]. Other studies have also confirmed T2DM as a risk factor for NAFLD. Conversely, patients with NAFLD have an increased risk of developing T2DM [13].

Currently, lifestyle modifications such as weight loss and physical activity are essential for all patients with NAFLD but are mostly not successful in the management of disease progression. Thus, there is an urgent need for approved medication therapy for NAFLD [4,5,14,15]. Ideal pharmacotherapy for NAFLD should decrease steatosis and inflammation, preferably liver fibrosis, while simultaneously improving insulin resistance, adiposity, and serum glucose levels [14]. There are currently numerous agents under clinical studies, and one of the leading candidates for Food and Drug Administration (FDA) approval are sodium–glucose cotransporter 2 (SGLT2) inhibitors [2,16,17]. Canagliflozin (Invokana), dapagliflozin, empagliflozin, ipragliflozin, tofogliflozin, and ertugliflozin, also known as “flozins”, are glucose-lowering agents with an insulin-independent mode of action [18,19,20,21,22]. They were FDA-approved as oral antidiabetic drugs to reduce glucose levels by the inhibition of glucose reabsorption in the proximal renal tubule and are, in general, not associated with a risk of hypoglycemia [23,24]. In general, SGLT2 inhibitors have a good safety profile, and the most frequent side effects reported are infection of the genitourinary tract and hypotension [16]. After gaining approval for T2DM, SGLT2 inhibitors were also approved for chronic kidney disease and heart failure in the non-diabetic population due to their beneficial effects on the cardiovascular and renal systems [5,25]. In general, the antifibrotic and anti-inflammatory effects of SGLT2 inhibitors have been proposed as a common mode of action for liver, kidney, and heart protection [26]. In this review, we provided an overview of the effects of SGLT2 inhibitors on NAFLD development with a focus on biomarkers that could be useful for early screening of NAFLD in patients with T2DM. 

### Effects of SGLT2 Inhibitors on Liver

The conversion of carbohydrates from the bloodstream into fatty acids and ultimately into triglycerides or other lipids is a highly coordinated process called de novo lipogenesis [27]. Catabolism and lipolysis in extrahepatic tissues, especially adipose tissue, result in the transfer of free fatty acids to the liver, where excess fat results in liver steatosis. Insulin regulates both de novo lipogenesis and lipolysis. Since insulin resistance is usually present in NAFLD patients, insulin is unable to adequately inhibit adipose lipolysis [28]. Since insulin resistance is usually present in NAFLD patients, insulin is unable to adequately inhibit adipose lipolysis [28]. Furthermore, adipose tissue affects NAFLD progression by producing hormones and cytokines, which contribute to the dysfunction of hepatocytes and by increasing liver uptake of lipids [29]. Lipoprotein lipase (LPL), the enzyme that regulates the rate of hydrolysis of VLDL and triglycerides, is also involved in NAFLD development due to upregulation [4]. Dysregulation of liver lipid metabolism is associated with metabolic diseases such as T2DM and fatty liver. Therefore, identifying biomarkers that contribute to the impairment of this process could be important in assessing liver steatosis [27,28].

There are several mechanisms by which SGLT2 inhibitors improve liver status and decrease liver fat in patients with T2DM. For example, inhibition of de novo lipogenesis by lowering glucose and insulin levels and increasing β-oxidation by elevating glucagon levels could shift the emphasis of metabolism from carbohydrate to fatty acid [5]. Some studies on SGLT2 inhibitor therapy in patients with or without T2DM have reported no significant correlation between changes in body mass index (BMI) and liver steatosis parameters, specifically the controlled attenuation parameter (CAP), an ultrasound method for measuring hepatic fat content. This confirmed that SGLT2 inhibitors have an impact on liver steatosis independent of weight loss [30,31]. Apart from decreasing oxidative stress caused by abnormally high glucose levels, SGLT2 inhibitors can downregulate pro-oxidants and free radical development and upregulate antioxidant molecules such as glutathione peroxidases (GSHs) and superoxide dismutases (SODs) [5,32,33,34].

The inhibition of inflammatory molecules such as interleukin-6 (IL-6) and tumor necrosis factor α (TNF-α) could be involved in SGLT2 inhibitors’ beneficial mechanisms in the liver [26]. Indeed, Bellanti et al. analyzed the impact of empagliflozin, dapagliflozin, and canagliflozin in 26 NAFLD patients and reported decreased levels of IL-6, IL-1ß, and TNF, as well as beneficial effects on liver steatosis and fibrosis [35]. 

Because ketone bodies have been detected in patients with T2DM treated with dapagliflozin and empagliflozin, it is considered that this class of medication triggers the disposal of fatty acids from liver lipid tissue, which are then oxidized to produce ketones due to a decrease in insulin and an increase in glucagon [36]. Another clinical study reported that ipragliflozin improved liver condition in a subgroup of 25 patients with hepatic steatosis, independent of body weight reduction [37]. Another study suggested that the beneficial effect of SGLT2 inhibitors on the liver triggered hepatic autophagy, a process of self-regeneration normally occurring in the fasting state [38,39]. Studies on animal models have shown that SGLT2 inhibitors induce autophagy by targeting the adenosine monophosphate-activated protein kinase-mammalian/mechanistic of rapamycin (AMPK-mTOR) signaling pathway [40,41]. It was found that during autophagy, dapagliflozin increased lipid oxidation and levels of amino acids leucine and valine, which could be future biomarkers of therapeutic efficacy with SGLT2 inhibitors [38]. 

An ongoing phase 3 clinical trial, DEAN (NCT03723252), aims to assess the efficacy and safety of dapagliflozin in 100 participants with T2DM and NASH. Outcomes for liver improvement and metabolic risk parameters will be studied [42,43]. Another ongoing phase 4 clinical trial (NCT05254626) aims to assess the effectiveness and safety of dapagliflozin compared with another antidiabetic drug, pioglitazone, in NASH patients with and without diabetes [43]. Interestingly, the recommendation to introduce SGLT2 inhibitors in NAFLD patients with T2DM is already included in guidelines developed by the Japanese Society of Gastroenterology in collaboration with the Japan Society of Hepatology [44]. 

## 2. Biomarkers

Microbiota and the gut–liver interaction play a very important role in liver homeostasis since most of the liver blood circulation comes from the gut. Leaky gut, altered microbiome, and impaired toxin clearance have been reported to be associated with NAFLD progression [5,9]. It is reported that patients with NAFLD consume significantly more fructose than controls [13]; fructose from soft drinks and *Ruminococcus* bacteria were found to be contributors to liver fibrosis [9]. Dapagliflozin improved microbial abundance and variety in mice with diabetes, while in control healthy mice, only a minor effect was observed, suggesting a potential beneficial effect of SGLT2 inhibitors on gut microbiota when associated with T2DM [45]. Furthermore, in a 3-month clinical trial, empagliflozin reduced the number of noxious bacteria *Escherichia-Shigella*, *Hungatella,* and *Bilophila* present in the gut microbiota of patients with T2DM [46]. Furthermore, SGLT2 inhibitors have been shown to induce increased short-chain fatty acid (SCFA)-producing bacteria, resulting in improved amino acid metabolism [47,48]. This is relevant because increased SCFAs have shown effects such as increased insulin sensitivity and a reduction in the hepatic fat storage in NAFLD. However, excessive SCFAs could also inhibit hepatic AMPK in the liver and increase the accumulation of hepatic FFAs [49,50]. Thus, in terms of microbiota and its metabolites as biomarkers, further research is required.

Alanine aminotransferase (ALT) and aspartate aminotransferase (AST) levels, due to hepatic inflammation, and gamma-glutamyl transferase (GGT) are the most used biomarkers for the assessment of liver disease. However, their values should be interpreted with caution as the patients with NAFLD can have normal levels. In addition, reduction in aminotransferase levels is not necessarily indicative of liver histology improvement [1,30,51]. However, SGLT2 inhibitors have effects on liver enzymes, as was reported in several studies on patients with NAFLD [20,52,53]. One prospective study in Japan on 43 T2DM patients with NAFLD reported decreased levels of GGT, AST, and ALT after 24 weeks of therapy with ipragliflozin [20]. Hayashi et al. assessed the effects of dapagliflozin on the lipid profile in 40 T2DM patients. Besides the inhibition of strong atherogenic sd low-density lipoprotein-C (sd LDL-C) and elevation of favorable high-density lipoprotein 2-C (HDL2-C), the study found significantly decreased plasma concentrations of both ALT and AST in dapagliflozin group 3 months after therapy initiation [52]. In another randomized clinical trial with dapagliflozin in T2DM patients with NAFLD, reduced GGT and ALT levels in the serum of the dapagliflozin-treated group were reported [53]. A meta-analysis and systematic review by Amjad et al., which included therapy with different SGLT2 inhibitors (ipragliflozin, empagliflozin, dapagliflozin, canagliflozin, and luseogliflozin) also demonstrated that SGLT2 inhibitors significantly improve liver status by lowering AST and ALT levels [54]. In Table 1, we provide an adapted summary of this study [54] updated with the most recently obtained evidence, as well as a detailed investigated biomarker list and their behavior in SGLT2 inhibitor treatment.

### 2.1. Non-Invasive Physical Methods

The most accurate method for NAFLD diagnosis is still liver biopsy, but it is invasive with potential adverse events [1,14]. As alternatives to biopsy, some non-invasive approaches to the diagnosis have been developed. Transient elastography (TE) and Magnetic Resonance Imaging (MRI) are the two most common techniques for NAFLD assessment [68]. 

Transient elastography (TE) is an ultrasound technique that uses FibroScan to measure at the same time two different parameters, CAP and Liver Stiffness Measurement (LSM), utilizing the same radiofrequency information [66,69,70]. CAP is a method that measures increased attenuation of the ultrasound beam when it crosses the liver tissue and is used for the evaluation of liver steatosis [69,71]. LSM is used for detecting liver fibrosis by measuring the velocity of the shear wave, which is mechanically produced when passing through the liver tissue [72]. Transient elastography was used in a 24-week randomized clinical trial to assess the effects of dapagliflozin on liver steatosis and fibrosis in patients with T2DM and NAFLD by measuring CAP and LSM values [53]. In the dapagliflozin group, the CAP value and visceral fat were significantly decreased, and the LSM value was significantly decreased in the severe fibrosis subgroup [53]. A double-blind clinical trial including 43 patients treated with empagliflozin and 47 with placebo demonstrated a significant decrease in LSM in the empagliflozin group (*p* = 0.001) and a significant decrease in the CAP parameter (*p* = 0.035) in a subgroup of patients treated with empagliflozin with more severe steatosis at baseline (CAP > 302 dB/m) [30]. Two other studies involving NAFLD and T2DM patients on empagliflozin therapy also reported significant decreases in CAP and LSM values [66,71]. A recent pilot study has demonstrated that the combination of empagliflozin and metformin compared to metformin monotherapy alleviated hepatic steatosis to a greater extent, showing significantly lower CAP value in patients with T2DM with dual therapy [73]. 

### 2.2. Potential Biomarkers of Steatosis

A number of serological biomarkers have been recommended for diagnosis and NAFLD treatment and monitoring [74]. The fatty liver index (FLI) is calculated based on triglycerides, GGT, BMI, and waist circumference values and is a commonly used biomarker to predict excessive liver fat content. A risk score of FLI > 60% is indicative of excessive hepatic fat, while an FLI < 30% rules it out [23,75].

**Figure 1:**
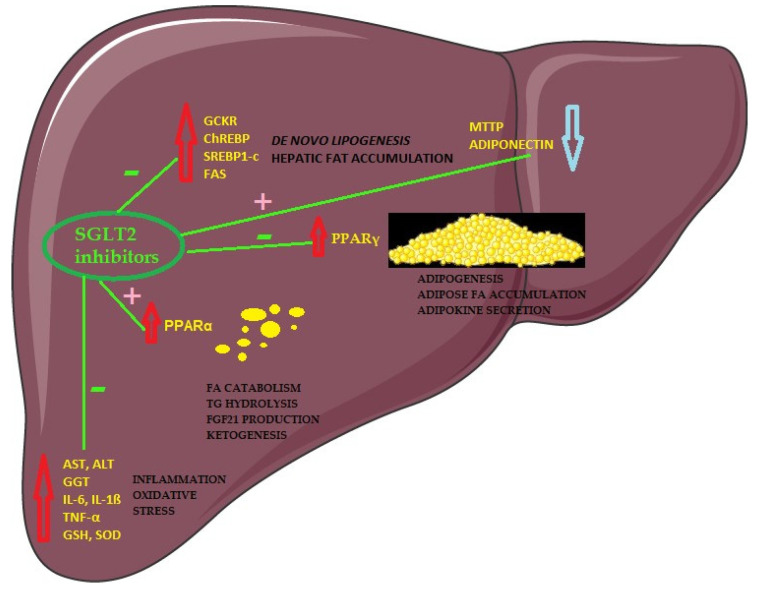
Effects of SGLT2 inhibitors on potential NAFLD biomarkers. SG GCKR: Glucokinase regulatory protein; ChREBP: Carbohydrate-responsive element-binding protein; SREBP1c: Sterol response element binding protein 1c; FAS: Fatty acid synthase; MTTP: Microsomal triglyceride transfer protein; PPARα: Peroxisome proliferator-activated receptor α; PPARγ: Peroxisome proliferator-activated receptor γ; FA: fatty acid; TG: Triglyceride; FGF21: Fibroblast growth factor 21; ALT: Alanine aminotransferase; AST: Aspartate aminotransferase; GGT: Gamma-glutamyl transferase; IL-6: Interleukin 6; IL-1β: Interleukin 1β; TNF-α: Tumor necrosis factor α; GSH: Glutathione peroxidase; SOD: Superoxide dismutase. Red arrow: upregulated biomarkers. Blue arrow: downregulated biomarkers. Green minus symbol: process leads to biomarkers inhibition. Pink plus symbol: process leads to biomarkers activation. Figure made with Servier Medical Art, https://smart.servier.com/ (accessed on 9 September 2023).

Peroxisome proliferator-activated receptors (PPARα, PPARβ/δ, and PPARγ) are a family of nuclear receptors acting as transcription factors [76]. Endogenous ligands that activate PPARs are lipids and therefore the dysregulation of PPARs is associated with metabolic disorders, which makes them especially suitable diagnostic and treatment targets (Figure 1) [76,77]. Although these three isoforms have different roles in liver lipid metabolism, they all have an anti-inflammatory effect on the liver [28]. Studies on patients have suggested that PPARs are involved in NAFLD development [28]. The downregulation of PPARγ was proposed as a mechanism of decreased liver adipogenesis in NAFLD in a study on mice treated with empagliflozin [78]. In the same study, empagliflozin improved mitochondrial β oxidation via the upregulation of PPARα, which was suppressed due to the high-fat diet [78]. 

Fibroblast growth factor-21 (FGF21) is a molecule present in many different organs, including the liver, and is induced by the transcription factor peroxisome proliferator-activated receptor α (PPARα) in a state of metabolic imbalance, such as fasting [79]. FGF21 has been detected as a potential biomarker of liver steatosis as its reduction is linked to the decrease in hepatic fat and enhancement of mitochondrial function [56]. Results of the 8-week RCT by Latva-Rasku et al. confirmed the reduction in liver fat and FGF21 in T2DM patients on dapagliflozin treatment [56]. Interestingly, a randomized controlled trial (RCT) reported that dapagliflozin monotherapy in T2DM patients was associated with a significant reduction in FGF21 levels, while no significant changes in placebo or dual therapy with dapagliflozin and omega-3 carboxylic acids groups were observed [57].

Hepatic de novo lipogenesis is controlled by glucose and insulin levels by sterol response element binding protein 1-c (SREBP1-c) and carbohydrate-responsive element-binding protein (ChREBP) proteins [80]. Sterol response element binding protein-1a, -1c, and -2 (SREBP-1a, -1c, and -2) are a family of integral proteins of endoplasmic reticulum which have a central role in controlling lipid metabolism [14]. SREBP1-c, a transcription factor regulated by insulin, is involved in de novo lipogenesis, and its overexpression in steatosis is linked to the deposition of triglycerides in the liver [14]. It was suggested that the sensitivity of the liver to insulin could be improved by damping lipogenesis by SREBP1 downregulation [81]. Several studies on animals have reported decreased expression of SREBP1 when SGLT2 inhibitor was administered [5]. Mice on a diet with high fat and empagliflozin for five weeks demonstrated lower levels of cholesterol, triglycerides, and SREBP-1c in comparison with the control group [82]. Empagliflozin reduced the expression of SREBP1 in another preclinical study in a rat model with no obesity or hyperglycemia, which suggested possible benefits in clinical practice in pre-diabetes and pre-obesity states [81]. 

Another participant in de novo lipogenesis is glucokinase regulatory protein (GCKR), and the substitution of proline to leucine P446L in its encoding gene is associated with NAFLD progression [7,83,84]. GCKRP variant P446L causes the upregulated activity of GCKR and, consequently, elevated glycolytic flux and carbohydrate-responsive element-binding protein (ChREBP) activity, which is a very powerful activator of de novo lipogenesis (Figure 1) [84]. ChREBP is a transcription factor in hepatocytes, which is also triggered by carbohydrate feeding [85]. The inhibition of ChREBP in mice with obesity and insulin resistance led to hepatic steatosis improvement, implicating ChREBP as a contributor to NAFLD development [14]. Interestingly, another study showed that the upregulation of ChREBP triggers lipogenesis but without affecting glucose metabolism and insulin resistance [86]. 

ChREBP controls microsomal triglyceride transfer protein (MTTP), which is located in the lumen of the endoplasmic reticulum of hepatic cells and is a crucial factor for the formation of lipoproteins with apolipoprotein-B and its delivery to different organs [80,87]. Because a lack of MTTP in animals and humans has been related to hepatic fat accumulation and hypolipidemia, it is thought that MTTP can contribute to NAFLD development [80,88]. In mice models with NASH, obesity, and diabetes type 2, Honda et al. reported that administration of ipragliflozin improved insulin resistance, triggering increased expression of MTTP and resulting in decreased lipotoxicity [89]. 

Fatty acid synthase (FAS), the main enzyme of de novo lipogenesis, is controlled by SREBP1 and is upregulated in steatosis (Figure 1) [4,5]. Furthermore, a correlation was found between FAS expression and the severity of liver damage. Therefore, this enzyme could be an important biomarker for the detection of liver steatosis [4]. When canagliflozin was administered in mice, Kawarasaki et al. demonstrated reduced FAS expression relative to fat mass, resulting in the absence of weight increase [90]. In a 24-week double-blind, placebo-controlled clinical trial, administration of canagliflozin to patients with poorly regulated T2DM demonstrated reduced accumulation of triglycerides within the liver [91]. 

Along with NAFLD development, apoptosis of hepatocytes results in the formation of many different substrates, including keratins (K-19) and cytokeratins 18 (CK-18), which can be detected in the blood of patients and are therefore proposed as potential biomarkers of NAFLD progression [4,92]. Antigen CK18-M65 measures intact and cleaved CK-18, and antigen CK18-M30 measures cleaved CK-18. The level of CK18-M30 was found to be much higher in patients with severe steatosis, so it was suggested that CK18-M30 could help in detecting patients with advanced hepatic steatosis [4]. In a double-blind, placebo-controlled clinical trial, patients with diabetes type 2 and NAFLD receiving only dapagliflozin had decreased both CK18-M65 and CK18-M30, which suggests a protective effect of SGLT2 inhibitors on hepatocytes in T2DM patients (Table 1) [57]. 

Adiponectin is an adipokine molecule predominantly produced by white adipose tissue, and its accumulation declines as fat mass increases. This bioactive protein is found to reduce liver steatosis and fibrosis, inflammation, and insulin resistance, and it is involved in NAFLD progression [93,94]. Low levels of adiponectin are found to be associated with a higher risk of T2DM, increased triglycerides, and BMI [95,96]. Some studies proposed that SGLT2 inhibitors could elevate adiponectin levels [97,98]. This was confirmed by an RCT, which reported increased levels of adiponectin and decreased liver fat in T2DM patients (n = 42) after 24 weeks of empagliflozin therapy [61]. 

#### Other Potential Biomarkers of Steatosis

A common contributor of liver steatosis worsening is the modified patatin-like phospholipase-domain containing 3 (PNPLA3) protein caused by methionine replacement at residue 148 by isoleucine [99]. Elevated levels of this mutation have been observed in those of Hispanic ethnicity, which might be the reason for a higher prevalence of NAFLD among this population [28]. The German Diabetes Study registered as a clinical trial with 917 patients demonstrated that patients with severe insulin resistance diabetes are more likely to be carriers of the PNPLA3 gene mutation and suggested that carriers of this mutation have lipotoxic environments that could promote insulin resistance and NAFLD development [100]. Mutated PNPLA3 protein has decreased hydrolase enzymatic function, resulting in anomalous lipid remodeling and excessive lipid liver deposition [101,102]. This mechanism was confirmed in a study by Lindén et al. on mice, which showed that decreased activity of the PNPLA3 variant leads to reduced liver fat [101,102].

The substitution of glutamate for lysine in the Human transmembrane 6 superfamily 2 (TM6SF2) mutant E167K gene is associated with incorrect folding and decomposition of the protein TM6SF2 located in the endoplasmic reticulum and endoplasmic reticulum–Golgi Apparatus (intermediate compartment) of liver, renal, and small intestine cells [28,103]. Protein TM6SF2 is an important player in lipid metabolism, and therefore, this mutation contributes to metabolic disease development and is found to be associated with hepatic steatosis progression [103]. A study on the finish population by Kim et al. demonstrated that TM6SF2 E167K reduced serum levels of VLDL/LDL and triglycerides and therefore cardiovascular risk but increased risk of hepatic steatosis and T2DM [104]. In 2020, Borén et al. published research on humans, which confirmed that TM6SF2 E167K fails to export VLDL and triglycerides from liver cells, which causes fat accumulation [105]. 

The systemic immune-inflammation index (SII), a newly introduced biomarker that indicates local immune reaction and overall systemic immune inflammation, is found to be associated with insulin resistance and could be useful in detecting NAFLD progression in diabetes patients. A study by Xie et al. in 2022 analyzed the association between SII and CAP and LSM in which it was found that SII value is significantly correlated with CAP, i.e., liver steatosis and not with LSM value, i.e., liver fibrosis [106]. This could be explained by the fact that one-third of patients had severe fibrosis without the presence of NASH [106]. Another cross-sectional study also found a significant correlation between SII and hepatic steatosis by measuring HIS (Hepatic steatosis index), which implies that SII could be an important biomarker for assessing hepatic steatosis [107].

### 2.3. Potential Biomarkers of Fibrosis

The level of hepatic fibrosis is the most critical prognostic factor for NAFLD mortality; therefore, fibrosis biomarkers were developed [2]. Of note, McGlinchey et al. investigated molecules, which are specific for each stage of NAFLD and found that the most important breaking point in the course of NAFLD progression is the transition from the F2 to F3 fibrosis stage because of the largest number of modified metabolites (n = 73) present [108]. A commonly used fibrosis biomarker is the Fibrosis-4 (FIB-4) score, which is calculated based on AST, ALT, platelet count values, and age. FIB-4 >2.67 rules in, and FIB-4 <1.30 rules out advanced fibrosis [23,70]. Long-term outcomes were assessed in a study by Arai et al., including 109 patients treated with SGLT2 inhibitors for three years, demonstrating reduced FIB-4 index, especially in patients with moderate and high risk of progressed fibrosis [26]. A superior impact on liver histology was recently proven in the RCT by Takeshita et al. due to a significantly decreased FIB-4 score in the tofogliflozin study group [22]. 

A more complex biomarker for the detection of advanced fibrosis is the NAFLD fibrosis score (NFS) resulting from albumin, age, BMI, platelet count, hyperglycemia/diabetes, and AST/ALT ratio values [70,109]. A retrospective study by Tada et al. demonstrated that both NFS and FIB-4 scores were associated with extrahepatic complications such as cardiovascular and cerebrovascular diseases, suggesting that in patients with NAFLD and T2DM, the risk of other complications and mortalities could be reduced by decreasing liver fibrosis [26,109]. 

Epigenetics are of great importance to NAFLD development, progression, and clinical pattern. A recent study regarding cardioprotective mechanisms of SGLT2 inhibitors found that empagliflozin prevented DNA methylation induced by high glucose. Another study associated histone post-translational modifications with SGLT2 inhibitors in diabetic kidney disease [110,111]. However, a scarcity of current literature regarding the effects of SGLT2 inhibitors on epigenetic mechanisms shows the need for further research regarding the effects of SGLT2 inhibitors on the epigenetics of NAFLD.

## 3. Future Directions

In general, the progression of NAFLD is usually asymptomatic, and there are no specific biomarkers available in clinical practice for NAFLD diagnosis [9]. The detection of NAFLD at an early stage is crucial for favorable disease outcomes by prevention of fibrosis. However, early detection of a highly prevalent disease requires biomarkers that accurately correlate with liver steatosis and fibrosis and are suitable for routine patient screening. Only with such tools can the evaluation of the efficacy of SGLT2 inhibitors and other novel agents be feasible. Recently, other target molecules for therapy have been described, including the modified patatin-like phospholipase-domain containing 3 (PNPLA3) protein, human transmembrane 6 superfamily 2 (TM6SF2), and systemic immune-inflammation index (SII). 

Currently available RCTs have shown SGLT2 inhibitors’ superior efficiency in the reduction in biomarkers such as AST, ALT, FIB-4, liver proton density fat fraction (PDFF), as well as reduced visceral and subcutaneous fat areas, compared to other antihyperglycemic agents (metformin, pioglitazone, sitagliptin, and glucagon-like peptide-1 receptor agonist, and glimepiride). No superior effect, however, was determined by measuring HbA1c, fasting plasma glucose (FPG), and the homeostasis model assessment of insulin resistance (HOMA-IR) [112].

## 4. Conclusions

Evidence to date suggests that SGLT2 inhibitors play a promising role in the prevention of NAFLD. However, whether the protective role in liver tissue is mediated by metabolic, direct effects of the drugs themselves, or a combination of these effects remains unclear. SGLT2 inhibitors lower blood glucose, body weight, and blood pressure on one hand and have beneficial effects on oxidative stress, chronic inflammation, β-oxidation, de novo lipogenesis, autophagy, and apoptosis of hepatocytes on the other. According to the clinical trials conducted so far, there appear to be some differences in the effects on the liver tissue between individual agents within the class. However, this could be a reflection of different study designs or specific mechanism(s) of action and their complementary effects on NAFLD. Therefore, the development of better biomarkers for the evaluation of NAFLD is needed to understand the underlying mechanism of action of SGLT2 inhibitors and other novel agents in the liver tissue. Although NAFL is considered a benign entity, it can progress to NASH, and at present, there is no simple method of predicting those who will progress. Because progression is silent and slow, the initiation of NASH is difficult to detect with current technology. It is for this reason that biomarker development is so important. Despite preclinical and clinical evidence that SGLT2 inhibitors may be helpful in the treatment of NAFLD, evidence of their efficacy and long-term outcomes for this indication is still lacking, and further large prospective double-blind and placebo-controlled studies are needed to ultimately confirm this evidence.

## Figures and Tables

**Table 1:** Randomized controlled trials (RCT) on patients with T2DM treated with SGLT2 inhibitors and observed effect on the biomarkers. Adapted and updated from Amjad et al. [54].

Author and Year	SGLT2 Inhibitor	Effect on Biomarker Investigated	Main Observed Effect on Liver
Takahashi et al., 2022 [55]	Ipragliflozin	Liver biopsy—reduced liver fat content	Improved liver fibrosis and reduced liver fat deposition (improved NASH)
Latva-Rasku et al., 2019 [56]	Dapagliflozin	FGF21 reduction	Reduced liver fat deposition
Eriksson et al., 2018 [57]	Dapagliflozin	MRI-assessed liver fat content (PDFF) CK18-M65 and CK18-M30 decreased	Reduced liver fat deposition
Kuchay et al., 2018 [58]	Empagliflozin	MRI-assessed liver fat content (PDFF) reduced	Reduced liver fat deposition
Shibuya et al., 2017 [59]	Luseogliflozin	CT obtained liver/spleen ratio reduced	Reduced liver fat deposition
Ito et al., 2017 [60]	Ipragliflozin	FIB-4 index improved	Improved liver fibrosis
Shimizu et al., 2019 [53]	Dapagliflozin	Transient elastography (FibroScan)—CAP parameters improved	Improved liver fibrosis (preventing progression)
Takeshita et al., 2022 [22]	Tofogliflozin	FIB-4 index improved	Improved liver fibrosis
Kahl et al., 2020 [61]	Empagliflozin	Adiponectin increased Uric acid decreased	Reduced liver fat deposition
Han et al., 2020 [62]	Ipragliflozin	Transient elastography (FibroScan)—CAP parameter and FLI improved	Reduced liver fat deposition
Hayashi et al., 2017 [52]	Dapagliflozin	AST, ALT, HDL, and LDL values decreased	Improved liver function: AST and ALT decreased. Suppression of potent atherogenic sd LDL-C and increased favorable HDL2-C
Harreiter et al., 2021 [63]	Dapagliflozin	MRI-assessed liver fat content (PDFF), fatty liver index (FLI) has improved	Reduced liver fat deposition
Bando et al., 2017 [64]	Ipragliflozin	ALT values decreased, Liver/spleen ratio increased	Improved liver function, reduced liver fat deposition
Kinoshita et al., 2020 [65]	Dapagliflozin	Liver/spleen ratio increased	Reduced liver fat deposition
Chehrehgosha et al., 2021 [66]	Empagliflozin	Transient elastography (FibroScan)—CAP parameters improved	Reduced liver fat deposition
Yoneda et al., 2021 [67]	Tofogliflozin	MRI-assessed liver fat content (PDFF) reduced	Reduced liver fat deposition

FGF21 = Fibroblast growth factor 21, MRI = Magnetic resonance imaging, PDFF = Proton density fat fraction, CT = Computerized tomography, CAP = Controlled attenuation parameter, AST = Aspartate aminotransferase, ALT = Alanine aminotransferase, HDL = High-density lipoprotein, LDL = Low-density lipoprotein, FLI = Fatty liver index.

## Data Availability

Data sharing is not applicable to this article as no new data were created or analyzed in this study.
